# Prediction of adjuvant chemotherapy response in triple negative breast cancer with discovery and targeted proteomics

**DOI:** 10.1371/journal.pone.0178296

**Published:** 2017-06-08

**Authors:** Angelo Gámez-Pozo, Lucía Trilla-Fuertes, Guillermo Prado-Vázquez, Cristina Chiva, Rocío López-Vacas, Paolo Nanni, Julia Berges-Soria, Jonas Grossmann, Mariana Díaz-Almirón, Eva Ciruelos, Eduard Sabidó, Enrique Espinosa, Juan Ángel Fresno Vara

**Affiliations:** 1 Molecular Oncology & Pathology Lab, Instituto de Genética Médica y Molecular-INGEMM, Hospital Universitario La Paz-IdiPAZ, Madrid, Spain; 2 Biomedica Molecular Medicine SL, Madrid, Spain; 3 Proteomics Unit, Center of Genomics Regulation (CRG), Barcelona Institute of Science and Technology (BIST), Barcelona, Spain; 4 Proteomics Unit, Universitat Pompeu Fabra (UPF), Barcelona, Spain; 5 Functional Genomics Centre Zurich, University of Zurich/ETH Zurich, Zurich, Switzerland; 6 Biostatistics Unit, Hospital Universitario La Paz-IdiPAZ, Madrid, Spain; 7 Medical Oncology Service, Instituto de Investigación Hospital Universitario Doce de Octubre-i+12, Madrid, Spain; 8 Medical Oncology Service, Hospital Universitario La Paz-IdiPAZ, Madrid, Spain; 9 CIBERONC. Instituto de Salud Carlos III, Madrid, Spain; University of South Alabama Mitchell Cancer Institute, UNITED STATES

## Abstract

**Background:**

Triple-negative breast cancer (TNBC) accounts for 15–20% of all breast cancers and usually requires the administration of adjuvant chemotherapy after surgery but even with this treatment many patients still suffer from a relapse. The main objective of this study was to identify proteomics-based biomarkers that predict the response to standard adjuvant chemotherapy, so that patients at are not going to benefit from it can be offered therapeutic alternatives.

**Methods:**

We analyzed the proteome of a retrospective series of formalin-fixed, paraffin-embedded TNBC tissue applying high-throughput label-free quantitative proteomics. We identified several protein signatures with predictive value, which were validated with quantitative targeted proteomics in an independent cohort of patients and further evaluated in publicly available transcriptomics data.

**Results:**

Using univariate Cox analysis, a panel of 18 proteins was significantly associated with distant metastasis-free survival of patients (p<0.01). A reduced 5-protein profile with prognostic value was identified and its prediction performance was assessed in an independent targeted proteomics experiment and a publicly available transcriptomics dataset. Predictor *P5* including peptides from proteins RAC2, RAB6A, BIEA and IPYR was the best performance protein combination in predicting relapse after adjuvant chemotherapy in TNBC patients.

**Conclusions:**

This study identified a protein combination signature that complements histopathological prognostic factors in TNBC treated with adjuvant chemotherapy. The protein signature can be used in paraffin-embedded samples, and after a prospective validation in independent series, it could be used as predictive clinical test in order to recommend participation in clinical trials or a more exhaustive follow-up.

## Introduction

Breast cancer is one of the leading causes of death among women in developed countries. Approximately 20% of the cases correspond to triple-negative tumours, i.e., those not expressing estrogen and progesterone receptors and with no HER2 over-expression. Triple-negative breast cancer (TNBC) is associated with a poor outcome when compared with other subtypes, due to its aggressive behavior and limited therapeutic options [1]. Adjuvant therapy for TNBC relies exclusively on chemotherapy, as hormonal agents and anti-HER2 therapy are no effective in this type of breast cancer. The standard chemotherapy used in this setting includes anthracyclines and taxanes, but even with the use of adjuvant therapy, relapse risk approaches 50% and it is even higher in patients with additional high-risk factors [2].

Moreover, the clinical and molecular heterogeneity within this TNBC subtype makes the treatment of these patients even more challenging as some patients never relapse, whereas others do suffer an early relapse from resistant tumors. Several gene expression profiling evidenced the existence of distinct molecular subgroups of TNBC [3–5]. So far, these molecular studies have not yet allowed the stratification of patients into categories with different prognosis and response to specific treatments. Also, no specific drugs have been developed for the specific treatment of TNBC, although clinical reports suggest a role for platinum compounds [6].

High-throughput technologies for the quantitation of biomolecules are providing a comprehensive view of the molecular changes in cancer tissues. These technologies allow for the simultaneous analysis of the whole genome, global gene and microRNA expression, DNA methylation and protein expression of tumor samples, and in conjunction with the development of bioinformatics tools, have revealed the molecular architecture of breast cancer [7–9]. Recently, two large-scale studies have addressed the structure of the TNBC genome, by means of next generation sequencing and have revealed a plethora of different genetic events occurring in TNBC. Moreover, the results of these studies also revealed the high diversity within this cancer subtype and that there are very few common genetics events in TNBC tumors; mainly a mutation of TP53 that occurs in approximately 80% of these tumors and loss of the tumor suppressor phosphatase PTEN occurring in 29%, with all other mutations occurring at a relatively low frequency [10, 11]. These observations are in agreement with results from other large-scale sequencing studies showing that cancers exhibit extensive mutational heterogeneity, with mutated genes varying widely across individuals [12].

The cellular genotype dictates the observed phenotype through the production of proteins, which, in turn, perform most of the reaction that occur in the cell. Proteomics analyses thus offer a means to measure the biological outcome of cancer-related genomic abnormalities, including expression of variant proteins encoded by mutations, protein changes driven by altered DNA copy number, chromosomal amplification and deletion events, epigenetic silencing, and changes in microRNA expression [13].

Mass spectrometry has become the method of choice for analyzing complex protein samples, and recent technological advances allow identifying thousands of proteins from tissue amounts compatible with clinical routine. Therefore, proteomics may become a new source of molecular markers with utility in the management of breast cancer patients and to facilitate clinical decisions in daily clinical practice. In the case of TNBC patients, the identification of protein signatures that define patient subgroups that need to be treated with a specific combination of drugs or alternative interventions is highly desirable. In this study, we identified a protein signature with a high prediction value in the response to adjuvant chemotherapy, and validated it in an independent cohort using quantitative targeted proteomics. Indeed, the described protein signature can predict adjuvant chemotherapy response in triple negative breast cancer samples, it is suitable to evaluate formalin-fixed, paraffin-embedded tumour samples, and therefore, it could be used to recommend participation in clinical trials or a more exhaustive follow-up in high-risk TNBC patients.

## Materials and methods

### Study design and sample description

The discovery cohort comprises twenty-six FFPE samples from patients diagnosed of triple negative breast cancer (TNBC) were retrieved from I+12 Biobank (RD09/0076/00118) and from IdiPAZ Biobank (RD09/0076/00073), both integrated in the Spanish Hospital Biobank Network (RetBioH; www.redbiobancos.es) between 1997 and 2004. The targeted proteomics cohort includes one hundred and fourteen samples from patients diagnosed of triple negative breast cancer were retrieved from I+12 Biobank (RD09/0076/00118) and from IdiPAZ Biobank (RD09/0076/00073), both integrated in the Spanish Hospital Biobank Network (RetBioH; www.redbiobancos.es) between 1997 and 2012. Sixty samples from I+12 Biobank were previously included in an analytical observational case–control study [14]. The histopathological features of each sample were reviewed by an experienced pathologist to confirm diagnosis and tumor content. Eligible samples had to include at least 50% of tumor cells.

### Ethics, consent and permissions

Written consent was provided by all patients participating in this study, and approval from the Ethical Committees of Hospitals Doce de Octubre and La Paz was obtained for the conduct of the study.

### Total protein extraction

Proteins were extracted from FFPE samples as previously described [15]. Briefly, FFPE sections were deparaffinized in xylene and washed twice with absolute ethanol. Protein extracts from FFPE samples were prepared in 2% SDS buffer using a protocol based on heat-induced antigen retrieval [16]. Protein concentration was determined using the MicroBCA Protein Assay Kit (Pierce-Thermo Scientific). Protein extracts (10 μg) were digested with trypsin (1:50) and SDS was removed from digested lysates using Detergent Removal Spin Columns (Pierce).

### Discovery mass spectrometry data acquisition

Samples were analyzed by liquid chromatography-mass spectrometry on a LTQ-Orbitrap Velos (Thermo Fischer Scientific, Bremen, Germany) coupled to NanoLC-Ultra system (Eksigent Technologies, Dublin, CA, USA) as previously described [17]. Peptide samples were further desalted using ZipTips (Millipore), dried, and solubilized in 15 μL of a 0.1% formic acid and 3% acetonitrile solution before MS analysis. Peptide separation was performed on a self-made C18 column (75μm×150mm, 3 μm, 200A) by a 5 to 30% acetonitrile gradient in 95 minutes. Each MS cycle consisted of a full scan MS spectra (300–1700) recorded at resolution of 30000 at 400 m/z followed by CID (collision induced dissociation) fragmentation on the twenty most intense signals. Charge state screening was enabled and singly charge states were rejected. Precursor masses selected for MS/MS were placed in a dynamic exclusion for 45s.

### Discovery mass spectrometry data analysis

Protein identification and quantification were performed using the Andromeda search engine and MaxQuant (version 1.2.7.4) [18]. Spectra were searched against a forward UniProtKB/Swiss-Prot database for human concatenated to a reverse decoyed fasta database and containing common protein contaminants. The precursor and fragment tolerances were set respectively to 20ppm and 0.5 Da, carbamidomethyl (C) was set as fixed modification while oxidation (M), deamidation (N, Q) and N-terminal protein acetylation were set as variable modifications. Enzyme specificity was set to Trypsin/P, allowing a minimal peptide length of 7 amino acids and a maximum of two missed cleavages. A maximum false discovery rate (FDR) of 0.01 for peptides and 0.05 for proteins was allowed.

Label free quantification was performed setting a 2 minutes window for match between runs. The protein abundance was calculated on the basis of the normalized spectral protein intensity (LFQ intensity). Quantifiable proteins were defined as those detected in at least 75% of TNBC samples showing two or more unique peptides. Only quantifiable proteins were considered for subsequent analyses. Protein expression data were log2 and missing values were replaced using data imputation for label-free data, as explained in [19], using default values. Finally, protein expression values were z-score transformed. Batch effects were estimated and corrected using ComBat [20].

All the shotgun mass spectrometry raw data files acquired in this study may be downloaded from Chorus (http://chorusproject.org) under the project name *Breast Cancer Proteomics*.

### Parallel reaction monitoring data acquisition

Between one and four unique peptides per protein were selected for quantification by parallel reaction monitoring (PRM), prioritizing those peptides that had been observed previously. The selected peptides were bought as isotopically labelled internal standard peptides (^13^C_6_,^15^N_2_-Lys and ^13^C_6_,^15^N_4_-Arg, Pepotec Peptides, Thermofisher Scientific) and they were spiked in the peptide mixture. The amount spiked-in per for each reference peptide was chosen based on the following criteria: i) to have an area as close to the endogenous peptide area as possible, and ii) to be in within the concentration range in which a linear response of the peptide was observed.

One third of each sample was analyzed using an Orbitrap Fusion Lumos (Thermo Fisher Scientific) coupled to an EASY-nanoLC 1000 UPLC system (Thermo Fisher Scientific) with a 50-cm C18 chromatographic column. Peptide mixes were separated with a chromatographic gradient starting at 5% B with a flow rate of 300 nL/min and going up to 22% B in 79 min and to 32% B in 11 min (Buffer A: 0.1% formic acid in water. Buffer B: 0.1% formic acid in acetonitrile). The Orbitrap Fusion Lumos was operated in positive ionization mode with an EASY-Spray nanosource at 1.4kV and at a source temperature of 275°C.

A scheduled PRM method was used for data acquisition with a quadrupole isolation window set to 1.4 m/z and MSMS scans over a mass range of m/z 340–950, with detection in the Orbitrap at a variable resolution depending on the peptide. PRM scans for heavy standards were performed at a resolving power of 15000 (at m/z 200); whereas PRM scans of endogenous peptides were performed at resolution 30000, 60000 or 120000 (at m/z 200) depending on its detectability and observed interferences in previous optimization experiments.

MSMS fragmentation was performed using HCD at 30 NCE, the auto gain control (AGC) was set at 50000 and the injection time (IT) was adjusted according to the transient length, with a maximum of 118 ms for 60000 resolution, and a minimum of 22 ms for 15000 resolution. The size of the scheduled window was 10 min and the maximum cycle time was 2.8 s. All data was acquired with XCalibur software v3.0.63. The Parallel Reaction Monitoring dataset is publicly available in the Panorama web server at https://panoramaweb.org/labkey/project/UPF%20-%20CRG/La%20Paz_TN_Breast_Cancer/begin.view?.

### Parallel reaction monitoring data analysis

Product ion chromatographic traces corresponding to the targeted precursor peptides were evaluated with Skyline software v2.5 based on i) traces co-elution, both in its light and heavy forms; and ii) the correlation between the relative intensities of the endogenous product ion traces, and their isotopically-labelled counterparts from the internal reference peptides.

For each monitored peptide a light-to-heavy ratio (L/H ratio = sum of product ion areas of the endogenous peptide/sum product ion areas from the reference peptide) was calculated per patient. Ratios were transformed to the logarithmic scale (log_2_) and the obtained values were used as proxy for protein amount.

### Prognostic models development and validation

Shotgun data were used to compute a statistical significance level for each protein based on a univariate proportional hazards model [21] with the aim of identifying proteins with an abundance level significantly related to the distant metastasis-free survival (DMFS) as described previously [22]. Briefly, proteins related to DMFS were filtered based on their p-values. Proteins with a p-value<0.01 were used to develop prediction models of recurrence risk using the supervised principal component method [23]. Additionally, we evaluated the correlation between the proteins to establish correlation groups and reduce the number of selected proteins to build the molecular signatures. Proteins with a Pearson correlation higher than 0.5 were grouped together and reduced profiles were designed including randomly proteins from different correlation groups. Leave-one-out cross-validation was used to evaluate the predictive accuracy of the profiles. The cutoff point was established *a priori* and to test the statistical significance, the p-value of the log-rank test statistic for the risk groups was evaluated using 1000 random permutations. Analyses were performed in BRB-ArrayTools v4_2_1. BRB-ArrayTools has been developed by Dr. Richard Simon and BRB-ArrayTools Development Team.

### Transcriptomics analyses

We used previously published transcriptomics array expression data of 1,296 primary breast carcinomas from two previously published works [24, 25]. Batch effects between data sets were estimated and corrected using ComBat [20]. After protein-to-gene ID conversion, all probes in dataset for each gene were retrieved. Probes with higher coefficient of variation were selected when multiple probes were found for a single gene. We selected estrogen receptor negative patients with TNBC characteristics, thus we excluded any patient showing an ESR1 relative expression above 12 and ERBB2 relative expression above 11.8, as described previously [26, 27]. Per-gene normalization within the validation cohorts was performed using median values obtained in the discovery cohort. Survival curves were then estimated [28]. Note that no clinical HER2 assessment was available for the transcriptomics samples and that the ERBB2 gene expression value was used for sample classification.

### Statistical analyses and software suites

Distant metastasis free survival (DMFS) was defined as the time between the day of surgery and the date of distant relapse or last date of follow-up. The independence of prognostic value of predictors when compared with clinical information was evaluated using multivariate Cox regression analyses. SPSS v16 software package, GraphPad Prism 5.1 and R v2.15.2 (with the *Design* software package 0.2.3) were used for all statistical analyses. All p-values were two-sided and p<0.05 was considered statistically signficant.

## Results and discussion

Triple-negative breast cancer (TNBC) accounts for one fifth of all breast cancers, and although they are usually treated with the administration of adjuvant chemotherapy after surgery, many patients have a relapse. Therefore, the main objective of this study was to identify proteomics-based biomarkers to stratify patients according to the benefits of the adjuvant chemotherapy, enabling the possibility to offer therapeutic alternatives to patients with predicted poor response to it.

### Patient’s characteristics

**Fig 1:**
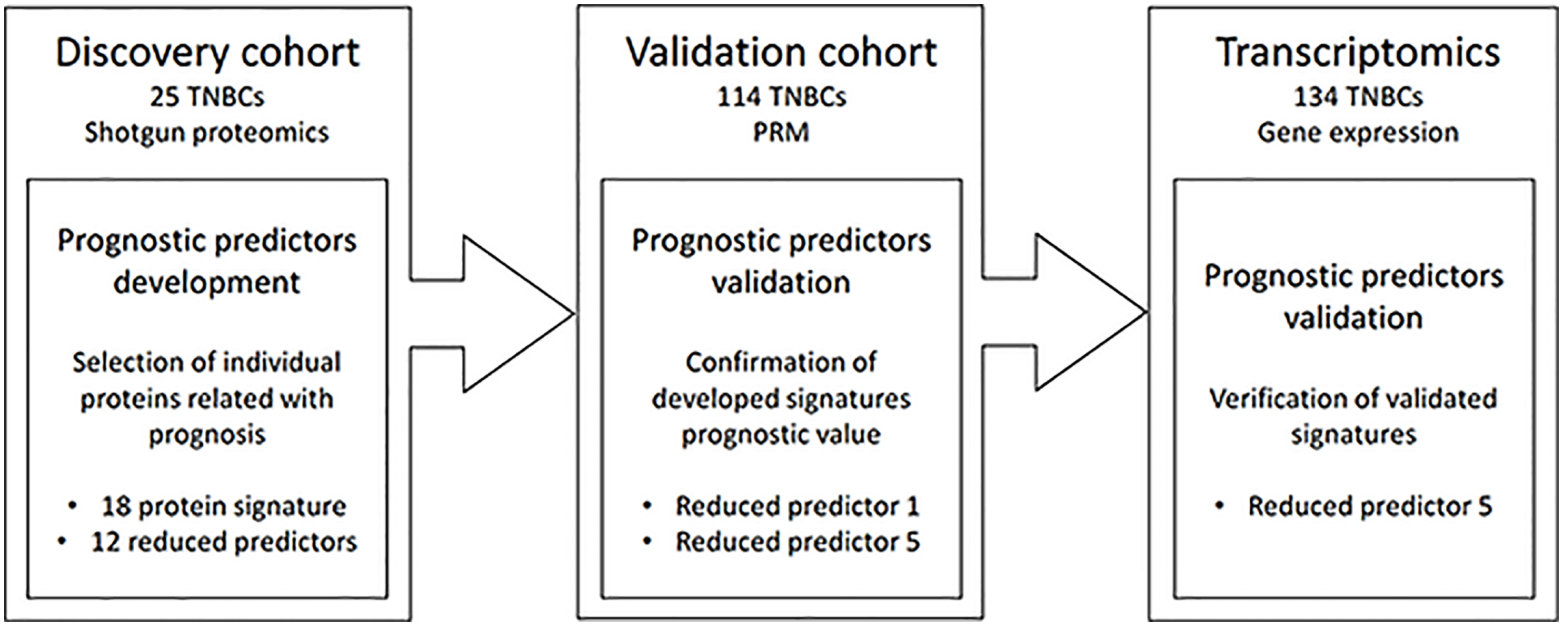
Study design. Chart of samples included and analysis performed in each cohort.

In order to identify prognostic biomarkers of the standard chemotherapy in TNBC patients, we included 25 TNBC patients to be in the discovery study, and 114 TNBC patients to be included in the targeted-proteomics study as an independent validation cohort. The clinical characteristics from all these patients are provided in Table 1. All included patients had node-positive disease; all of the tumors were negative when tested for hormonal receptors using immunohistochemistry and Her2 amplification using immunohistochemistry and fluorescent *in situ* hybridization when needed. Adjuvant chemotherapy was used in all cases (either anthracycline-based or not). In the discovery patient cohort, the median follow-up of all patients was 8.14 years (range: 1.24–12.95) and 9 patients had relapse events. In the validation cohort, median follow-up of all patients was 5.29 years (range: 0.47–11) and 56 patients had relapse events. Adjuvant chemotherapy was used in all patients (either anthracycline-based or not) except in four cases Study design is schematized in Fig 1.

**Table 1:** Clinical characteristics of the patients included in the study.

	Discovery cohort	Validation cohort
**Age at diagnosis (median)**	61.2 (37–78)	57 (25–89)
**Age at diagnosis (mean)**	58.5	58.9
**Tumor Size**		
**T1**	4 (19%)	51 (35.6%)
**T2**	19 (73%)	109(76.2%)
**T3**	2 (8%)	7(4.89%)
**T4**	0 (0%)	8(5.59%)
**Multifocal**	0 (0%)	1(0.69%)
**Tumor Grade**		
**G1**	0 (0%)	4(2.79%)
**G2**	4 (16%)	22(15.38%)
**G3**	19 (76%)	112(78.32%)
**Unknown**	2 (8%)	5(3.49%)
**Lymph node status**		
**N0**	0 (0%)	75(52.44%)
**N1**	17 (68%)	41(28.67%)
**N2**	8 (32%)	10(6.99%)
**N3**	0 (0%)	14(9.79%)
**Nx**	0 (0%)	3(2.09%)
**Chemotherapy**		
**No Antraciclines**	11 (42%)	19(16.7%)
**Antraciclines**	12 (46%)	62(54.3%)
**Antraciclines + taxanes**	2 (12%)	9(7.9%)
**Unknown**	0(0%)	20(17.6%)
**No**	0(0%)	4(3.5%)
**Median follow-up (years)**	8.14 (1.24–12.95)	5.29 (0.47–11)
**Relapse events (%)**	9(36%)	56(49%)

Clinical criteria are provided according to TNM classification (http://www.cancer.gov/cancertopics/pdq/treatment/breast/healthprofessional/page3). Tumor grade is the description of a tumor based on how abnormal the tumor cells and the tumor tissue look under a microscope.

### Molecular characterization of TNBC samples by discovery proteomics

Initially, we set up to perform discovery mass spectrometry-based proteomics of the collected 25 FFPE breast cancer samples to identify potential protein candidates that could be used as prognostic biomarkers to chemotherapy response of TNBC patients. Tissue samples were prepared for mass spectrometry analysis with trypsin digestion, following a previously-reported method that exhibit a high reproducibility for these type of samples [23]. Protein abundance data resulting from the mass spectrometry shotgun data acquisition constituted our “*discovery dataset*”. One sample was excluded from the study because it was considered an outlier as it did not reach the “mean minus twice the standard deviation”-threshold in the number of unique peptides identified. A total of 3,095 protein groups were identified using the Andromeda database search engine (S1 Table, of which 1,064 presented at least two unique peptides and were detectable in at least 75% of the samples (S2 Table)). Protein label-free quantification was further performed using MaxQuant LFQ values.

In order to identify proteomics-based biomarkers to stratify patients according to the benefits of adjuvant chemotherapy, we performed a survival analysis using the proteins quantified in the discovery dataset and related them with distant metastasis free survival with the Survival Analysis Tool from BRB-ArrayTools. We found that 18 out of 1064 proteins were significantly associated with distant metastasis-free survival (DMFS) of patients in the discovery dataset (Table 2)

**Table 2:** Proteins significantly associated with distant metastasis free survival.

UniProtKB accession numbers	Uniprot ID	Protein name	Gene Symbol	Hazard ratio	P value
**O43175**	SERA_HUMAN	D-3-phosphoglycerate dehydrogenase (3-PGDH) (EC 1.1.1.95)	PHGDH PGDH3	0.689	0.001
**O75323**	NIPS2_HUMAN	Protein NipSnap homolog 2 (NipSnap2) (Glioblastoma-amplified sequence)	GBAS NIPSNAP2	1.830	0.001
**P05091**	ALDH2_HUMAN	Aldehyde dehydrogenase, mitochondrial (EC 1.2.1.3) (ALDH class 2) (ALDHI)	ALDH2 ALDM	0.423	0.002
**P05161**	ISG15_HUMAN	Ubiquitin-like protein ISG15 (Interferon-induced 15 kDa protein) (Interferon-induced 17 kDa protein) (IP17) (Ubiquitin cross-reactive protein) (hUCRP)	ISG15 G1P2 UCRP	0.500	0.002
**P07996**	TSP1_HUMAN	Thrombospondin-1	THBS1 TSP TSP1	0.649	0.002
**P14317**	HCLS1_HUMAN	Hematopoietic lineage cell-specific protein (Hematopoietic cell-specific LYN substrate 1) (LckBP1) (p75)	HCLS1 HS1	0.379	0.003
**P15153**	RAC2_HUMAN	Ras-related C3 botulinum toxin substrate 2 (GX) (Small G protein) (p21-Rac2)	RAC2	0.423	0.003
**P18085**	ARF4_HUMAN	ADP-ribosylation factor 4	ARF4 ARF2	3.754	0.003
**P20340**	RAB6A_HUMAN	Ras-related protein Rab-6A (Rab-6)	RAB6A RAB6	0.493	0.004
**P28065**	PSB9_HUMAN	Proteasome subunit beta type-9 (EC 3.4.25.1) (Low molecular mass protein 2) (Proteasome subunit beta-1i) (Really interesting new gene 12 protein)	PSMB9 LMP2 PSMB6i RING12	0.758	0.005
**P53004**	BIEA_HUMAN	Biliverdin reductase A (BVR A) (EC 1.3.1.24) (Biliverdin-IX alpha-reductase)	BLVRA BLVR BVR	0.674	0.006
**P62873**	GBB1_HUMAN	Guanine nucleotide-binding protein G(I)/G(S)/G(T) subunit beta-1 (Transducin beta chain 1)	GNB1	0.703	0.006
**Q09666**	AHNK_HUMAN	Neuroblast differentiation-associated protein AHNAK (Desmoyokin)	AHNAK PM227	1.614	0.006
**Q15046**	SYK_HUMAN	Lysine—tRNA ligase (EC 6.1.1.6) (Lysyl-tRNA synthetase) (LysRS)	KARS KIAA0070	0.672	0.008
**Q15181**	IPYR_HUMAN	Inorganic pyrophosphatase (EC 3.6.1.1) (Pyrophosphate phospho-hydrolase)	PPA1 IOPPP PP	2.184	0.008
**Q9BUP0**	EFHD1_HUMAN	EF-hand domain-containing protein D1 (EF-hand domain-containing protein 1) (Swiprosin-2)	EFHD1 SWS2 PP3051	0.265	0.009
**Q9GZZ9**	UBA5_HUMAN	Ubiquitin-like modifier-activating enzyme 5 (Ubiquitin-activating enzyme 5) (ThiFP1) (UFM1-activating enzyme) (Ubiquitin-activating enzyme E1 domain-containing protein 1)	UBA5 UBE1DC1	0.316	0.009
**Q9NR31**	SAR1A_HUMAN	GTP-binding protein SAR1a (COPII-associated small GTPase)	SAR1A SAR1 SARA SARA1	0.222	0.009

These 18 proteins are significant with p< 0.01 in the univariate test.

Proteomics candidates found in the discovery dataset were also checked in a transcriptomics expression data from 134 triple negative breast cancer samples from two publicly available dataset [24, 25]. To this purpose, per-gene normalization within the validation cohorts was performed. It has been already demonstrated that mRNA levels largely reflect the respective protein levels [29, 30]. Consequently, the intersection between proteomic data sets and other genome-wide data sets often allows robust cross-validation [31, 32].

### Identification and validation of prognostic protein based signatures in TNBC patient samples

Protein abundances derived from shotgun mass spectrometry data in the discovery dataset were then used to identify protein combinations with prediction value of distant metastasis free (DMFS) survival after standard chemotherapy. The validation of the prediction value of each proposed protein combination was validated in an independent 114 TNBC patients cohort performing protein quantitation with parallel reaction monitoring approach (PRM), a targeted proteomics approach that enables the quantification of a set of preselected peptides of interest (S3, S4, S5 and S6 Tables). Moreover, proteomics candidates found in the discovery dataset were further assessed in transcriptomics expression data from 134 triple negative breast cancer samples from two publicly available dataset.

Initially, the identified 18 proteins to be significantly associated with DMFS were initially used to build a protein predictor of DMFS containing all 18 proteins. The cutoff threshold value was bounded *a priori* to split the population with a 50:50 distribution between low and high distant metastasis risk. DMFS at 5 years was 100% for patients defined as low-risk by the prognostic profile versus 25% for patients defined as high-risk (hazard ratio (HR) = 16.36, p<0.0001). However, the prognostic value of this signature could not be validated neither using PRM data from the validation cohort nor using the publicly available transcriptomics dataset. In the PRM validation cohort, DMFS at 5 years was 59.8% for patients defined as low-risk by the prognostic profile versus 56.6% for patients defined as high-risk when used a 50:50 cutoff value (HR = 1.065, p = 0.78). In the transcriptomics verification, when using a 50:50 cutoff, DMFS at 5 years was 71.3% for patients defined as low-risk by the prognostic profile versus 66.5% for patients defined as high-risk (HR = 1.309, p = 0.38).

We then explored the possibility of developing a protein combination using a reduced number of proteins, as the incorporation of redundant information may reduce the chances of finding a valid predictor [28]. Towards this direction, we established three groups of proteins based on the correlation of their expression abundance patterns and one or two proteins belonging to different correlation groups were randomly included to build predictors that included three to seven proteins. Again, a 50:50 distribution between low and high distant metastasis risk was set *a priori* to obtain a cutoff threshold value. Twelve protein combinations were built and they all exhibited a significant prognostic value in our discovery dataset (S1 Fig and S7 Table).

Using the protein abundances derived from the PRM analysis of the 114 TNBC tumor samples, we could validate two out of twelve reduced predictors, which also showed a significant prognostic value in an independent cohort of patients (Table 3). Predictor P1 showed a significant prognostic value using a 70:30 distribution between low and high risk patients. DMFS at 5-years was of 65.6% in the low-risk group and 29.92% at high-risk group (HR = 2.577, p = 0.0002). Predictor *P5* showed a significant prognostic value using a 70:30 distribution between low and high risk patients. DMFS at 5-years was of 63.54% in the low-risk group and 39.99% at high-risk group (HR = 2.322, p = 0.0142). Moreover, predictor *P5* also showed a significant prognostic value when compared with tumor size and lymph node status using multivariate Cox regression analyses (S8 and S9 Tables), and when used to predict the behavior of the patients analyzed in the transcriptomics dataset.

**Table 3:** DMFS prediction of the two reduced predictors tested in the publicly available transcriptomics dataset.

Reducedpredictor	ProteinID	DMFS^$^(low risk)	DMFS^$^(high risk)	HR(95%CI)	p	DMFS^$^(low risk)	DMFS^$^(high risk)	HR (95%CI)	p
		70:30 cutoff				50:50 cutoff			
**Predictor_1PRM validation**	P53004P05161P28065O75323	67.1%	29.9%	3.277(1.740–6.172)	>0.01	73.9%	37.2%	3.094(1.906–5.540)	>0.01
**Predictor_5PRM validation**	P53004P20340P15153Q15181	63.5%	39.9%	1.774(1.057.– 3.453)	0.01	61.7%	48.8%	1.327(0.787–2.246)	0.15
		Defined cutoff				50:50 cutoff			
**Predictor_1Transcriptomics**	P53004P05161P28065O75323	72.0%	68. %	1.311(0.647–2.668)	0.45	67.7%	71.0%	0.907(0.495–1.66)	0.75
**Predictor_5Transcriptomics**	P53004P20340P15153Q15181	80.9%	66.6%	1.837(0.844–3.998)	0.13	76.4%	63. %	1.888(1.027–3.468)	0.041

^$^DMFS is calculated at five years

**Fig 2:**
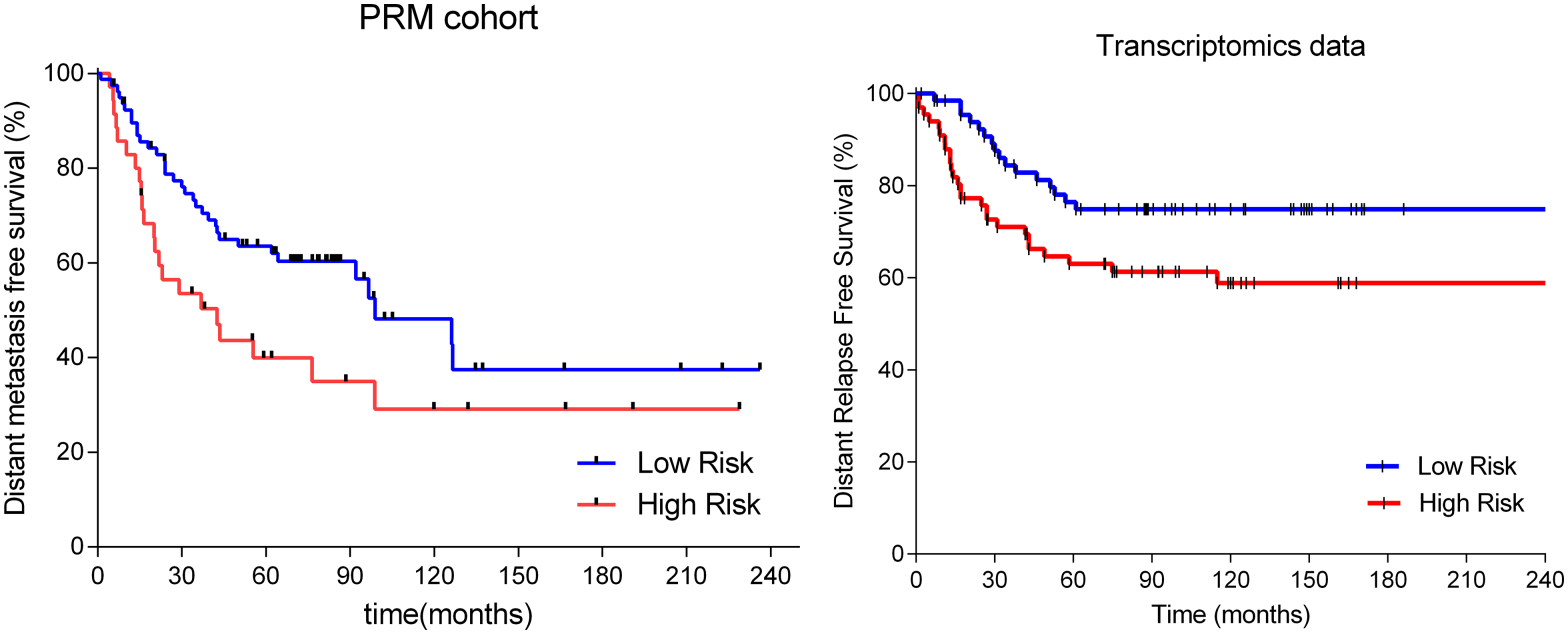
Survival analysis of reduced profile 5 in the PRM validation cohort and in the trasncriptomics orthogonal verification.

Finally, we also checked the performance of the reduced predictors *P1* and *P5* in the two publicly available transcriptomics datasets. In these data, predictor *P1* showed no prognostic information, whereas predictor *P5* showed a DMFS in the low-risk group over 80% using the test set defined cutoff thresholds, but they assigned less than 20% of the patients to this group. However, this last results leaves too many patients who do not relapse in the high-risk group, and thus, we tested a 50:50 cutoff threshold in this predictor. When a 50:50 cutoff threshold was used DMFS at five years in the publicly available transcriptomics dataset was 78.0% for low-risk patients versus 61.4% (HR = 2.888, p = 0.041) (Table 3 and Fig 2).

Predictor *P5* includes peptides from proteins RAC2, RAB6A, BIEA and IPYR. RAC2 is a member of the Ras superfamily of small guanosine triphosphate (GTP)-metabolizing proteins. It has been proposed that protein RAC2 might have a role in the regulation of the actin cytoskeleton during breast cancer metastasis [33]. RAC2 is also involved in both PLD-induced cell invasion [34] and oncogenic KIT-induced neoplasms [35], and its under-expression has been related to invasive and metastatic competence in human cancer [36]. BIEA, the protein encoded by the biliverdin reductase A (BLVRA) gene, belongs to the biliverdin reductase family members, which catalyze the conversion of biliverdin to bilirubin in the presence of NADPH or NADH. It also works as a dual-specificity kinase (S/T/Y), and activates the MAPK and IGF/IRK receptor signal transduction pathways [37, 38]. BIEA plays a pivotal role in the development of multidrug resistance in human HL60 leukemia cells [39], and itis included among the 50 genes that compose the PAM50 gene signature for classifying “intrinsic” subtypes of breast cancer [40].

RAB6A is a member of the RAB family, which belongs to the small GTPase superfamily. This protein is located at the Golgi apparatus, which regulates protein-trafficking. RAB6A is a potential target of both miR-21 and miR-155, known to be deregulated [41] and be correlated with a poor prognosis in breast cancer [42–44], which supports our findings. Additionally, RAB6A showed an increased expression in the HER-2/neu breast cancer subgroup [45].

Finally, IPYR is a cytosolic inorganic pyrophosphatase, codified by the PPA1 gene. PPA1 expression is significantly higher in many tumors, especially those of lung and ovarian origin. Expression of IPYR is heterogeneous in breast cancer cells [46] and the knockdown of PPA1 shows a decreased colony formation and viability of MCF7 cells [47]. Additionally, pyrophosphatase overexpression has been associated with cell migration, invasion, and poor prognosis in gastric cancer [48].

## Conclusions

High-throughput proteomics can be used to identify subgroups with different prognosis among patients with TNBC and to derive signatures with a combination of multiple proteins that enable patient stratification. Defining multi-gene or multi-protein predictors for prognosis increases their accuracy, reproducibility and robustness, which are highly desirable features in clinical diagnostic and prognostic tools. Towards this direction, Liu and colleagues developed a 11-protein signature in early triple-negative breast cancer [49] which showed a prognostic value in lymph node negative patient who had not received systemic adjuvant therapy. The protein signature was validated in an independent dataset using a cutoff determined from the ROC curve of the training set to ensure high-sensitivity and specificity. However, for validation purposes it is usually important that cutoff thresholds of a risk score be defined in advance [50]. Other authors have defined prognostic and predictive signatures in TNBCs using gene expression measurement techniques [4, 51, 52].

In the present work, we described the first protein-based signatures to predict adjuvant chemotherapy response in triple negative breast cancer samples. Several protein predictors were derived from a shotgun mass spectrometry-based discovery dataset and their performance was further validated in an independent patient cohort using targeted proteomics (parallel reaction monitoring). Our protein signatures were derived from routinely processed FFPE samples on a population of TNBC patients treated with adjuvant chemotherapy, which is closer to the clinical reality. Within these context, predictor *P5* that includes peptides from proteins RAC2, RAB6A, BIEA and IPYR, emerged as the best predictor when accounting both the discovery and the validation proteomics datasets. Moreover, its performance was also confirmed in a publicly available transcriptomics dataset, which exemplify the robustness of the described predictor and its applicability to patient-derived transcriptomics data that might be already collected.

Although our findings require prospective validation in independent series for routine clinical application, our work demonstrates the potential of proteomics to assist oncologists to make clinical decisions regarding patient treatment; e.g., patients classified with the low-risk group by the identified protein signature need to be treated with standard chemotherapy, whereas those classified with the high-risk group should be offered clinical trials with new drugs and an intensive follow-up program.

## Supporting information

S1 Fig Kaplan-Meier graphs of reduced profiles. (TIF)

S1 Table Shotgun proteomics LFQ values. (TXT)

S2 Table Log_2_ transformed and normalized protein expression data. (TXT)

S3 Table Sample and patient codes of PRM analyses. (XLSX)

S4 Table Scheduled PRM Method for Orbitrap Fusion Lumos. (XLSX)

S5 Table Product ion area for quantified endogenous and isotopically-labelled peptides. (XLSX)

S6 Table Log2 ratio of the areas of the quantified endogenous and isotopically-labelled peptides. (XLSX)

S7 Table Survival analysis of reduced profiles in the discovery cohort. (DOCX)

S8 Table Multivariate Cox regression model in discovery cohort. T: tumor size, N: lymph node status, HR: Hazard Ratio.
(DOCX)

S9 Table Multivariate Cox regression model in targeted-proteomics cohort. T: tumor size, N: lymph node status, HR: Hazard Ratio.
(DOCX)

## Abbreviations

DMFS: distant metastasis-free survival
FDR: false discovery rate
FFPE: formalin-fixed paraffin-embedded
HR: hazard ratio
TNBC: triple negative breast cancer
