# Supplementary figures and images for: Prediction of adjuvant chemotherapy response in triple negative breast cancer with discovery and targeted proteomics
Source: PLoS One. 2017 Jun 8;12(6):e0178296. doi: 10.1371/journal.pone.0178296 (PMC5464546; doi:10.1371/journal.pone.0178296)

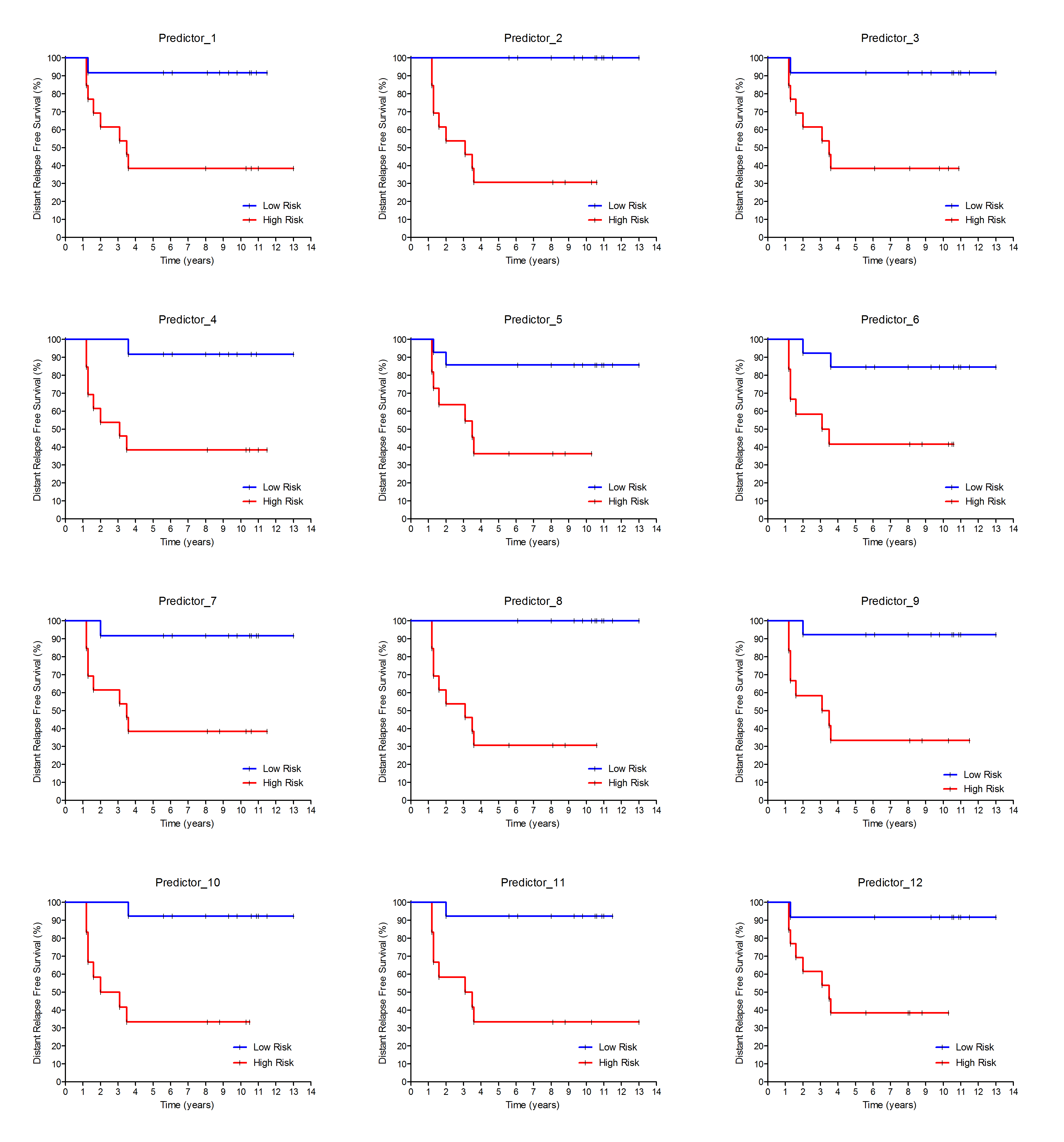

Supplement: S1 Fig — (TIF) [file pone.0178296.s001.tif]
