# Supplementary material for: Prediction of adjuvant chemotherapy response in triple negative breast cancer with discovery and targeted proteomics
Source: PLoS One. 2017 Jun 8;12(6):e0178296. doi: 10.1371/journal.pone.0178296 (PMC5464546; doi:10.1371/journal.pone.0178296)
Supplement: S7 Table — (DOCX) [file pone.0178296.s008.docx]

Supplementary Table 3

| Reduced predictor | Proteins included | DMFS 5 years (low risk) | DMFS 5 years (high risk) | HR  (95% CI) | p-value | AUC |
| --- | --- | --- | --- | --- | --- | --- |
| Predictor_1 | P53004  P05161  P28065  O75323 | 91.6 % | 38.5 % | 6.116  (1.613 -23.20) | 0.0078 | 0.831 |
| Predictor_2 | P05091  P14317  P18085  P15153  Q09666 | 100 % | 30.8 % | 11.75  (3.040 - 45.40) | 0.0004 | 0.849 |
| Predictor_3 | P14317  P18085  P28065  Q9BUP0 | 91.6 % | 38.5 % | 6.116  (1.613 -23.20) | 0.0078 | 0.894 |
| Predictor_4 | P05091  Q15046  P62873  P07996 | 91.6 % | 38.5 % | 7.301  (1.889 - 28.21) | 0.0039 | 0.784 |
| Predictor_5 | P53004  P20340  P15153  Q15181 | 85.7 % | 36.4 % | 5.806  (1.478 - 22.80) | 0.011 | 0.806 |
| Predictor_6 | P14317  O43175  P62873 | 84.6 % | 41.6 % | 5.264  (1.349 - 20.53) | 0.017 | 0.824 |
| Predictor_7 | P14317  P18085  P28065 | 91.6 % | 38.5 % | 6.466  (1.698 - 24.62) | 0.0062 | 0.74 |
| Predictor_8 | P14317  P28065  Q9NR31 | 100 % | 30.8 % | 11.75  (3.040 - 45.40) | 0.0004 | 0.796 |
| Predictor_9 | P53004  P20340  P15153  P14317  P18085  P28065 | 92.3 % | 33.3 % | 8.526  (2.186 - 33.26) | 0.0024 | 0.877 |
| Predictor_10 | P53004  O43175  P62873  P18085  P05091 | 92.3 % | 33.3 % | 9.941  (2.493 - 39.65) | 0.0011 | 0.794 |
| Predictor_11 | P53004  P20340  P15153  P14317  P18085  P28065  Q9NR31 | 92.3 % | 33.3 % | 8.526  (2.186 - 33.26) | 0.0024 | 0.903 |
| Predictor_12 | P53004  P20340  P15153  Q9NR31 | 91.7 % | 38.5 % | 6.116  (1.613 - 23.20) | 0.011 | 0.83 |
