# Supplementary material for: Prediction of adjuvant chemotherapy response in triple negative breast cancer with discovery and targeted proteomics
Source: PLoS One. 2017 Jun 8;12(6):e0178296. doi: 10.1371/journal.pone.0178296 (PMC5464546; doi:10.1371/journal.pone.0178296)
Supplement: S8 Table — T: tumor size, N: lymph node status, HR: Hazard Ratio. (DOCX) [file pone.0178296.s009.docx]

***Supplementary Table 8: Multivariate Cox regression model in discovery cohort.***

T: tumor size, N: lymph node status, HR: Hazard Ratio.

|  | | | |
| --- | --- | --- | --- |
|  | p | HR |  |
| T | .541 | 1.399 |  |
| N | .871 | .863 |  |
| predictor P5 | .022 | 6.729 |  |
